# High-Quality Genome Assembly of *Chrysaora quinquecirrha* Provides Insights Into the Adaptive Evolution of Jellyfish

**DOI:** 10.3389/fgene.2020.00535

**Published:** 2020-06-04

**Authors:** Wangxiao Xia, Haorong Li, Wenmin Cheng, Honghui Li, Yajing Mi, Xingchun Gou, Yaowen Liu

**Affiliations:** ^1^Shaanxi Key Laboratory of Brain Disorders, Institute of Basic Translational Medicine, Xi’an Medical University, Xi’an, China; ^2^Center for Ecological and Environmental Sciences, Northwestern Polytechnical University, Xi’an, China; ^3^Key Laboratory of Animal Gene Editing and Animal Cloning in Yunnan Province, Yunnan Agricultural University, Kunming, China

**Keywords:** jellyfish, genome, evolution, assembly, adaptation

## Abstract

Jellyfish, such as *Chrysaora quinquecirrha*, hold an important evolutionary position and have great ecological value. However, limited genomic resources are currently available for studying their basic genetic and development processes. Here, we *de novo* assembled the first high-quality reference genome of *C. quinquecirrha*, and successfully annotated 21,606 protein-coding genes. Codon usage analysis identified the frequent use of low-GC-content codons during protein-coding gene translation. Analysis of the relative evolution rate indicated that jellyfish had a faster evolution rate than sea anemones but slower rate than the species in Hydra. Phylogenetic analysis with two other species of jellyfish indicated that *Aurelia aurita* and *Nemopilema nomurai* have a closer relationship with each other than with *C. quinquecirrha*, with divergence from their common ancestor occurring ≈475.7 million years ago. Our study not only showed the genomic characteristics and molecular adaptive evolution of *C. quinquecirrha*, but also provides valuable genomic resources for further study on complex developmental processes and environmental adaptations.

## Introduction

Jellyfish of the phylum Cnidaria are lower invertebrate gelatinous zooplankton. They are considered one of the most primitive branches of the “tree of life” and thus one of the earliest ancestral species (Lucas and Dawson, 2014; Ou et al., 2015). Jellyfish are generally regarded as diploblastic animals which has endoderm and ectoderm but lack of mesoderm. They further possess a primitive reticular nervous system that controls muscle contractions and that consists of nerve cells connected through nerve projections to form a neural network (Kellie et al., 1980; Ou et al., 2015). In addition, several jellyfishes have no real eyes, but a relatively unsophisticated visual organ made up of fibrous photoreceptors that sense changes in external light (Martin, 2002; Nilsson, 2004; Suga et al., 2008).

The increase in the frequency of jellyfish blooms in recent years has also drawn attention due to their potential to transform marine ecosystems (Brotz et al., 2012; Duarte et al., 2013; Pitt and Lucas, 2014). Jellyfish are carnivores that feed on zooplankton, fish eggs, and larvae, and can greatly impact plankton populations when blooms occur (Lilley et al., 2009; Acuña et al., 2011; Marques et al., 2015; Nagata and Morandini, 2018). However, very few animals feed on jellyfish, and thus the large amounts of carbon sequestered within their bodies is not transferred within the food web. As such, jellyfish are often considered to be at the energy terminal and can seriously affect the marine environment and ecological processes (Pitt et al., 2005; Turk et al., 2008; Condon et al., 2011; Oguz et al., 2012). Several studies have reported that jellyfish blooms have an important relationship with their own physiological reproduction (Hamner and Dawson, 2009; Kawahara et al., 2012; Schiariti et al., 2014). The life cycle of most jellyfish species consists of alternating modes of reproduction between generations, i.e., polyp and medusa (Schiariti et al., 2014; Lee et al., 2017). Under suitable environments, medusae reproduce sexually to produce hydroids, which, in turn, reproduce asexually to produce ephyrae. Finally, ephyrae develop into new medusae, allowing jellyfish to bloom in a cycle of reproduction and growth (Johnson et al., 2001; Fuchs et al., 2014; Schiariti et al., 2014).

There are about 2,000 kinds of jellyfish worldwide, including ∼200 species in the phylum Scyphozoa (Daly et al., 2007; Chang E. S. et al., 2015; Costa et al., 2015). As a representative of Scyphozoa, *Chrysaora quinquecirrha* is one of the most well-known and well-studied jellyfish species, with high ecological value along the Atlantic coast of the USA and Gulf of Mexico (Cones and Haven, 1969; Calder, 1974; Johnson et al., 2001; Meredith et al., 2016; Bayha et al., 2017). Genomics analysis can help clarify the genetic information and evolutionary origin of different species (Chang E. S. et al., 2015; Kim et al., 2019). Although several jellyfish species’ genome has been studied (Ryan et al., 2013; Gold et al., 2019; Jiang et al., 2019; Kim et al., 2019; Leclère et al., 2019; Ohdera et al., 2019), the genome of *C. quinquecirrha* has not yet been assembled or analyzed. In this study, we *de novo* assembled the first high-quality reference genome of *C. quinquecirrha*, and successfully obtained 21,606 protein-coding genes. Furthermore, based on genomics analysis, we elucidated the evolutionary history and genetic changes of *C. quinquecirrha* relative to closely related species. This study not only provides valuable information on the evolutionary status and genetic changes of jellyfish, but also provides a foundation for future studies on the development and evolutionary origin of multi-cellular animals.

## Materials and Methods

### Sampling and Sequencing

Fresh muscle samples of jellyfish (*Chrysaora quinquecirrha*) were dissected and prepared for DNA and RNA extraction. For genome sequencing, we extracted high-quality DNA using a Qiagen Blood & Cell Culture DNA Mini Kit for Nanopore long-read (Oxford Nanopore, United Kingdom) and Illumina short-read (insert size: ∼250 bp; Pair-end 150 bp) sequencing. For RNA sequencing, RNA was extracted from muscle samples by Trizol (Invitrogen) according to the manufacturer’s instructions and sequenced on the Illumina platform (insert size: ∼250 bp; Pair-end 150 bp).

### Data Filtering

Nanopore long reads were filtered by the mean quality value of each read with in-house Perl scripts, and only reads with a quality value > seven were retained. For Illumina short reads, including genome and transcriptome sequencing data, we used the same standards for quality control. Specifically, any read with more than 50% low-quality bases or 10% unknown bases were filtered, and adaptor sequences and duplicated reads produced during polymerase chain reaction (PCR) were also removed. Then, all the remaining sequencing reads were used for further analysis.

### Genome Characteristic Estimation

Genome characteristics were evaluated using the genomic short reads based on the k-mer method. The reads were divided into a 17-bp length with 1-bp walking length. The k-mer frequency/number in each k-mer depth was then calculated, and genome size was estimated by the total k-mer number and peak k-mer frequency of 17-mer.

### Genome Assembly and Quality Evaluation

Although the Nanopore sequencing reads have a length advantage over the Illumina sequencing reads, they show low accuracy. In this study, we corrected the sequencing errors in the Nanopore reads using NextDenovo.^1^ The corrected Nanopore long reads were then used for genome assembly with WTDBG (v2.1) (Ruan and Li, 2019) and parameters: -p 15 –k 7 –AS 2 –E 1 –s 0.05 –L 5000. We further corrected the sequencing errors in the genome assembly with Racon (v1.2.1) and Pilon (v1.21) (Walker et al., 2014). Then, we mapped the corrected Nanopore reads to the assembled contigs by Minimap (v2.9) with parameters: -a –x map-ont –k 17. The haplotigs and low coverage contigs were removed by Purge_haplotigs (v1.1.1). The SSPACE-LongRead (v1.1) was used anchor the contigs to scaffolds, and Gapcloser (v1.10) was used to fill the gaps in the scaffold assembly. To evaluate the integrity of the assembled genome, we aligned all high-quality Illumina sequencing reads to the genome using BWA (v0.7.12) (Li and Durbin, 2009). The integrity of the protein-coding regions in the genome was evaluated by the mapping ratio of transcripts using BLAT (v34) (Kent, 2002).

### Repetitive Element Annotation

To identify more complete repetitive sequences in the genome, we used RepeatModeler (v1.0.4)^2^ for *de novo* prediction of repetitive sequences, and RepeatMasker (open-4.0.7) (Bedell et al., 2000) for repetitive sequence prediction using both the RepeatModeler results and the public repbase library. RepeatProteinMask (open-4.0.7) was used for predicting transposable elements (TEs) at the protein level, and tandem repeats were analyzed by Tandem Repeat Finder (v4.04) (Benson, 1999).

### Annotation of Protein-Coding Genes

To exclude the influence of repetitive sequences in the assembled genome during the coding-gene annotation process, we masked all repetitive sequences and then employed coding-gene annotation using different strategies. We first *de novo* predicted the protein-coding genes using AUGUSTUS (v2.5.5) (Stanke and Waack, 2003). We next downloaded several published gene sets, including *Hydra vulgaris* (GCF_000004095.1), *Stylophora pistillata* (GCF_002571385.1), *Acropora digitifera* (GCF_000222465.1), *Nematostella vectensis* (GCA_000209225.1), *Exaiptasia pallida* (GCF_001417965.1), *Aurelia aurita* (GCA_004194415.1), and *Renilla muelleri* (GigaDB) (Putnam et al., 2007; Chapman et al., 2010; Shinzato et al., 2011; Baumgarten et al., 2015; Voolstra et al., 2017; Gold et al., 2019; Jiang et al., 2019). We then aligned all gene sets to the annotated *C. quinquecirrha* protein sequences by tblastn (*e* = 10e-5) and predicted the gene structure using Genewise (v2-2-0) (Birney et al., 2004). After this, we *de novo* assembled the transcripts using Bridger software (r2014-12-01) (Chang Z. et al., 2015) and used them for coding-region prediction. Lastly, we merged the above results using EvidenceModeler (v1.1.1) (Haas et al., 2008). To better understand the biological functions of the annotated genes, we aligned them to public databases, including the InterPro, Gene Ontology (GO), SwissProt, TrEMBL, and Kyoto Encyclopedia of Genes and Genomes (KEGG).

### Orthologous Genes

Orthologous gene identification was conducted among these 10 species, including *H. vulgaris*, *S. pistillata*, *A. digitifera*, *N. vectensis*, *E. pallida*, *A. aurita*, *R. muelleri*, *Nemopilema nomurai*, *Echinococcus granulosus*, and *C. quinquecirrha*. Protein-coding genes among these species were used for orthologous relationship determination with OrthoMCL (v2.0.9) (Li et al., 2003). From these results, the 1:1 single-copy genes among the 10 species were selected and used for specific analyses.

### Phylogenetic Relationship Determination and Divergence Time Estimation

To determine the phylogenetic relationships among the 10 species, we aligned the 1:1 single-copy genes using MUSCLE (v3.8.31) (Edgar, 2004). We then conducted phylogenetic analysis in RAxML (v8.2.10) (Stamatakis, 2014), with *E. granulosus* as the outgroup. Divergence time analysis was conducted using the MCMCtree program in PAML (v4.8) (Yang, 2007), and fossil records downloaded from the TIMETREE website^3^ were used for result calibration.

### Codon Usage and Relative Evolution Rate

The protein-coding genes were used for codon usage analysis with CodonW (1.4.4; -all_indices -c_type 2 -f_type 4 -nomenu -nowarn -totals) (Peden, 1999) and in-house Perl scripts. Relative evolution rates among different species were analyzed using LINTRE (njboot -d7; tpcv -d7 -o 1) (Takezaki et al., 1995) and MEGA software (Tajima’s Relative Rate Test) (Kumar et al., 2018), with *C. quinquecirrha*as the reference species and *E. granulosus* as the outgroup.

### Expansion and Contraction Analysis of Gene Families

Gene family expansion and contraction were analyzed using CAFÉ (v3.1) (De Bie et al., 2006) with three input files: i.e., (1) Phylogenetic relationships were determined by RAxML (Stamatakis, 2014), (2) Divergence time was determined by MCMCtree (Yang, 1997), and (3) Orthologous relationships were determined by OrthoMCL (Li et al., 2003). The expanded or contracted gene families in the three jellyfish species were selected for further analysis.

## Results

### High-Quality Reference Genome Assembly of *C. quinquecirrha*

**FIGURE 1:**
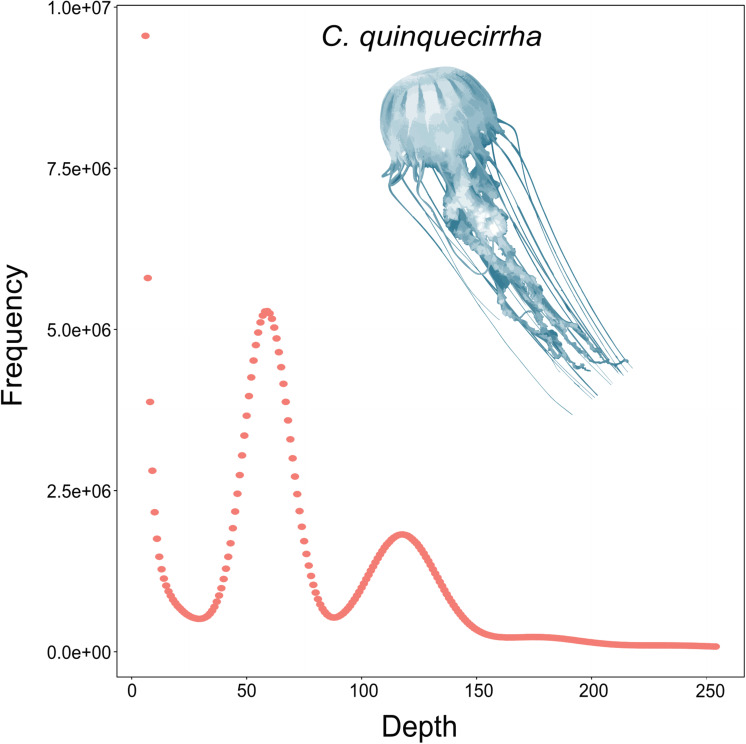
Genomic survey of *C. quinquecirrha*.

To acquire the *C. quinquecirrha* reference genome, we first extracted DNA from muscle tissue for genome sequencing. We then obtained 51.46 Gb of Illumina short reads (Supplementary Table S1) and determined the genome characteristics (e.g., genome size, repetitive sequence content, heterozygosity ratio). We used the 17-mer method and found the *C. quinquecirrha* genome is very complex, with high heterozygosity and repeat sequences, and has a genome size of 330.67 Mb (Figure 1). To better complete the assembly, we next sequenced the genome on the Nanopore platform (Promethion, Oxford Nanopore Technology) and acquired 81.12 Gb of high-quality reads, accounting for ∼245 genome coverage (Supplementary Table S2). We then corrected the potential sequencing errors in the Nanopore long reads with Nextdenovo^1^ and assembled the genome with WTDBG (Ruan and Li, 2019). To further improve the base accuracy of the acquired genome, we polished the assembly by the Nanopore and Illumina sequencing data with Racon and Pilon, respectively. Then, the haplotigs and low coverage contigs were removed by Purge_haplotigs, scaffolded by SSPACE-LongRead, and gap-filled by Gapcloser software. Finally, we got a genome assembly with the contig N50 and scaffold N50 length of 230.04 and 733.65 Kb, respectively (Table 1). This could comparable with previously published high-quality genomes of closely related species (Supplementary Table S3). We then aligned the genome assembly with the core gene set in BUSCO (Simão et al., 2015), with nearly 80% of the conserved gene set among eukaryotes were found in the genome (Supplementary Table S4). We also mapped the Illumina short reads (Supplementary Table S1) and *de novo* assembled transcripts (with 99.6% BUSCO values) (Supplementary Tables S5–S7) and found that most could be successfully aligned to the genome assembly (Supplementary Tables S8, S9). Thus, we obtained a high-quality (high accuracy and connectivity) reference genome for *C. quinquecirrha*.

**TABLE 1:** Statistics of *C. quinquecirrha* genome assembly.

Term	Contig		Scaffold	
		
	Length (bp)	Number	Length (bp)	Number
N90	29,200	1,911	66,354	666
N80	60,058	1,158	205,342	365
N70	93,806	731	395,469	249
N60	147,444	457	555,468	178
N50	230,037	281	733,647	125
Max length (bp)	3,764,053	–	4,015,784	–
Total size (bp)	320,327,670	–	336,819,409	–
Total number (> 100 bp)	–	4,456	–	2,496
Total number (> 10 kb)	–	3,227	–	1,691

### Genome Annotation of *C. quinquecirrha*

**FIGURE 2:**
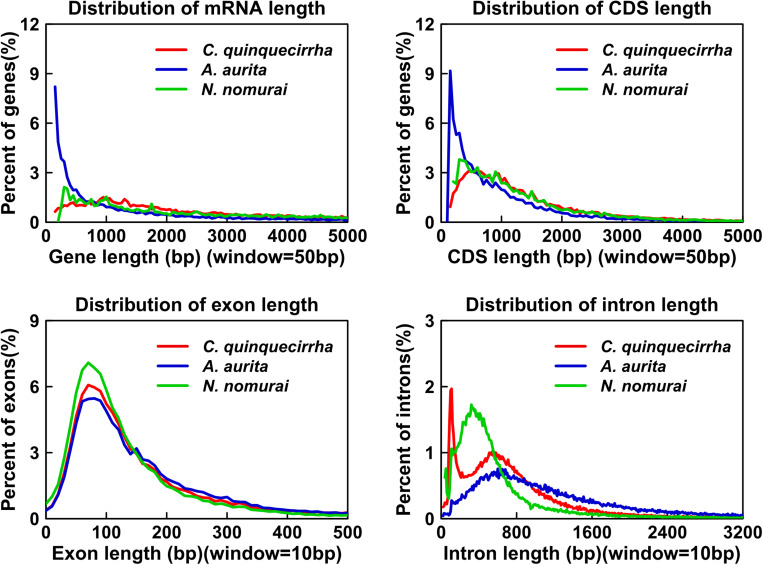
Statistics and comparison of protein-coding genes.

Genome annotation, which can greatly help to improve our understanding of species, was used in the current study. By combining the repetitive annotation results from several repeat annotation software, we successfully acquired 149.86 Mb of repetitive sequences, accounting for 44.49% of the assembled genome (Supplementary Table S10). Furthermore, 40.78% of the genome consisted of transposable elements (TEs) (Table 2), which could be divided into long terminal repeats (LTR, 4.07%), DNA elements (6.27%), short interspersed nuclear elements (SINE, 0.46%), and long interspersed nuclear elements (LINE, 5.70%). After repetitive sequence annotation, we first masked all repetitive sequences in the genome and employed protein-coding gene annotation by combining *de novo* prediction, homolog-based annotation, and transcript-based annotation. Finally, we merged the gene sets from the different strategies using EvidenceModeler software (Haas et al., 2008) and acquired 21,606 high-quality protein-coding genes in the *C. quinquecirrha* genome (Supplementary Table S11). We then compared and evaluated the annotation quality of the gene sets and found the quality to be comparable to that of closely related species (Figure 2). To better understand the biological functions of these genes, we performed functional annotation by aligning the protein sequences to the public databases, including GO, KEGG, InterPro, SwissProt, and TrEMBL. Most protein-coding genes could be found in the databases (Table 3), suggesting that we acquired a high-quality protein-coding gene set of the *C. quinquecirrha* genome. In addition to the coding-genes have key roles in biological processes, studies have shown that many non-coding RNAs (ncRNAs) also participate in and regulate many important physiological processes (Wilusz et al., 2009; Rinn and Guttman, 2011; Wang and Chang, 2011; Ulitsky and Bartel, 2013). Therefore, we systematically annotated and identified the ncRNAs in the *C. quinquecirrha* genome, including 7,833 tRNAs, 857 rRNAs, 745 snRNAs, and 50 miRNAs. These results could help clarify the functions of ncRNA in *C. quinquecirrha* (Table 4).

**TABLE 2:** Transposable elements in *C. quinquecirrha* genome.

Type	Repbase TEs		TE protiens		*De novo*		Combined TEs	
				
	Length (bp)	Percentage in genome	Length (bp)	Percentage in genome	Length (bp)	Percentage in genome	Length (bp)	Percentage in genome
DNA	709,874	0.21	1,888,178	0.56	18,705,956	5.55	21,120,760	6.27
LINE	796,165	0.24	4,927,606	1.46	14,347,687	4.26	19,209,048	5.70
SINE	2,252	0.00	0	0.00	1,545,091	0.46	15,46,202	0.46
LTR	1,631,255	0.48	6,442,822	1.91	6,651,643	1.97	13,718,366	4.073
Other	8,929,221	2.65	394,266	0.12	13,124,857	3.90	15,697,233	4.66
UnKnown	37,621	0.01	0	0.00	69,551,725	20.65	69,589,343	20.66
Summary*	11,859,774	3.52	13,648,690	4.05	121,407,295	36.05	137,342,709	40.78

The summary* line shows the non-redundant transposable elements of the above six categories. The percentage number in this table was rounded with two decimal places.

**TABLE 3:** Functional annotation of protein-coding genes in *C. quinquecirrha* genome.

Database	Number	Percentage (%)
InterPro	13798	63.86
GO	9733	45.05
KEGG	11049	51.14
SwissProt	11973	55.42
TrEMBL	16785	77.69

**TABLE 4:** ncRNA annotation in *C. quinquecirrha* genome.

Type	Sub-types	Copy (w)	Average length (bp)	Total length (bp)	Percentage of genome
miRNA	–	50	128.82	6,441	0.001912
tRNA	–	7,833	75.72	593,093	0.176086
rRNA	rRNA	857	129.46	110,948	0.03294
	18S	184	184.24	33,900	0.010065
	28S	331	164.62	54,488	0.016177
	5.8S	17	68	1,156	0.000343
	5S	325	65.86	21,404	0.006355
snRNA	snRNA	745	160.61	119,652	0.035524
	CD-box	16	104.75	1,676	0.000498
	HACA-box	0	0	0	0
	Splicing	729	161.83	117,976	0.035026

### Repetitive Sequence Expansions in *C. quinquecirrha* Genome

**FIGURE 3:**
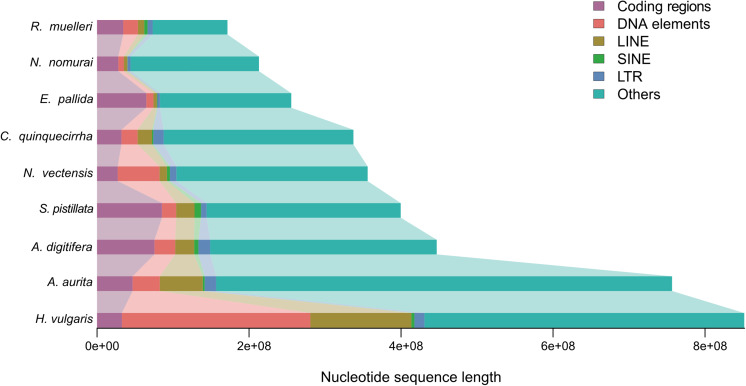
Genome size comparison among species.

**FIGURE 4:**
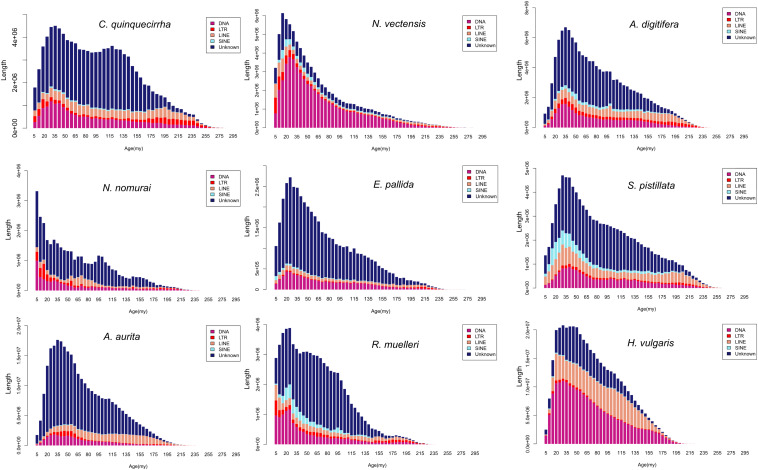
Repetitive sequence expansion and insertion history among species.

The genome sizes varied widely among Cnidaria species, especially in jellyfish (Figure 3), but the reasons of the genome expansion remain unclear. Thus, we compared the content of TEs (including LINE, SINE, LTR, and DNA elements) and coding regions among these species, and found that the main contributors to jellyfish genome expansion were non-coding regions (e.g., TEs), rather than coding regions (Figure 3). Further analysis demonstrated that the largest expansion of TEs in the *C. quinquecirrha* genome was that of SINE, with 5.40 times more SINE than that found in *N. nomurai* (Figure 3). To clarify the insertion history of TEs in jellyfish, we further analyzed the expansion history and found that TE expansion occurred within ∼235 million years in the three jellyfish species studied, and that the different insertion/expansion rates caused the differences in genome size (Figure 4).

### Orthologous Genes and Gene Family Analysis

**FIGURE 5:**
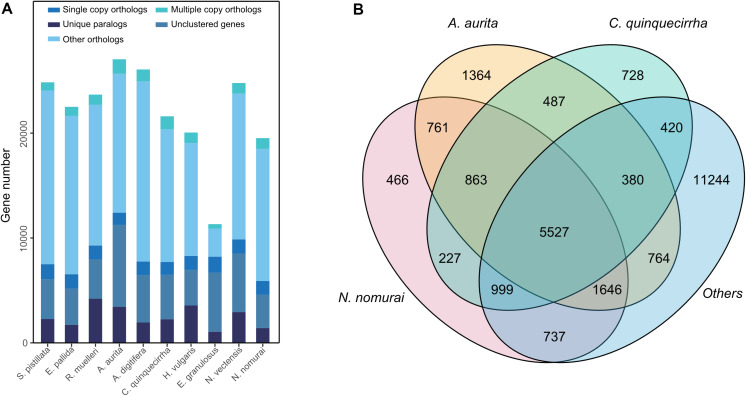
Gene family analysis of these species. **(A)** Gene family statistics among species. **(B)** Shared or specific gene families among species.

For comparative genomics analysis, we first downloaded the protein-coding genes of several species, including *N. vectensis*, *A. digitifera*, *N. nomurai*, *E. pallid*, *S. pistillata*, *A. aurita*, *R. muelleri*, *E. granulosus*, and *H. vulgaris*. Cluster relationships among these protein-coding genes were then determined by OrthoMCL (Li et al., 2003). We identified 26,613 gene families among the 10 species and 459 1:1 single-copy genes (Figure 5A and Supplementary Table S12). To identify gene families that may contribute to their unique characteristics, we conducted gene family analysis for the three jellyfish species relative to the other seven species, and found 728 gene families that specifically existed in jellyfish (Figure 5B), suggesting the possible unique functions of these genes in jellyfish. We further conducted enrichment analysis of the specific gene families. Results showed enrichment in several biological processes, including Hedgehog signaling pathway (*P* = 0.001342) and TGF-beta signaling pathway (*P* = 0.012341; Supplementary Table S13), thus suggesting that these genes contributed to unique development and adaptive evolution.

### Phylogenetic Relationships, Divergence Time, and Gene Family Expansion and Contraction

**FIGURE 6:**
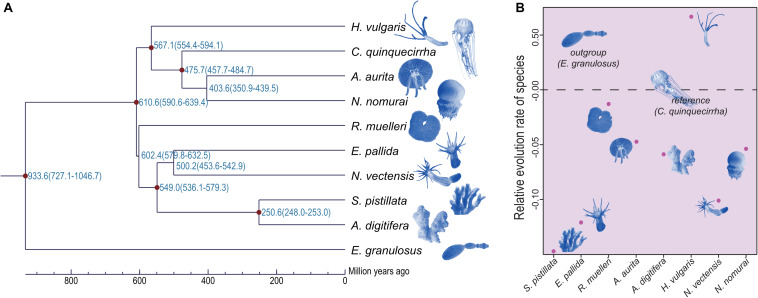
Phylogenetic relationship and relative evolution rate analyses of these species. **(A)** Phylogenetic relationship among these species. **(B)** Relative evolution rate among these species.

Although the phylogenetic relationships of jellyfishes and their closely related species have been investigated (Kayal et al., 2018), the whole-genome level phylogenetic tree of *C. quinquecirrha* and other species have not been studied. Here, we analyzed their phylogenetic relationships using RAxML software (PROTGAMMAJTT model; 100 bootstrap replicates) (Stamatakis, 2014). Results showed that *A. aurita* and *N. nomurai* has a close relationship than with *C. quinquecirrha* (Figure 6A). Divergence time analysis indicated that *A. aurita* and *N. nomurai* diverged 403.6 million years ago (Mya), and *C. quinquecirrha* diverged with the common ancestor of *A. aurita* and *N. nomurai* 475.7 Mya (Figure 6A and Supplementary Table S14). Furthermore, we conducted gene family analysis and identified 85 expanded and 64 contracted gene families (*P* < 0.05) in jellyfish. Functional analysis identified 2 GO and 30 KEGG terms were enriched in expansion, respectively (Supplementary Tables S15, S16). We found that biological processes, such as dorso-ventral axis formation (*P* = 0.000264635), fatty acid degradation (*P* = 0.002908315), and Notch signaling pathway (*P* = 0.005299582), were expanded in jellyfish relative to closely related species (Supplementary Table S16), suggesting genes in these families may have important functions in jellyfish.

### Codon Usage and Relative Evolution Rate

Jellyfishes are relatively ancient and simple multi-cellular organisms. Whether they exhibit similar codon usage with other species remains unclear. Here, we performed codon usage analysis in jellyfish and found it has no obvious differences with closely related species (Supplementary Table S17). Based on manually checking, we identified the frequent low-GC-content codons usage in *C. quinquecirrha*, including Glu and Asp (Supplementary Table S18), suggesting that low-energy codons are more commonly used in *C. quinquecirrha*. We next analyzed the relative evolution rate of species, with *C. quinquecirrha* as the reference and *E. granulosus* as the outgroup. Results indicated that jellyfish have a faster evolution rate than sea anemones but a slower rate than the species in Hydra (Figure 6B and Supplementary Tables S19, S20), suggesting different survival pressures and environmental adaptations during their evolutionary history.

## Discussion

We *de novo* assembled the first high-quality reference genome of *C. quinquecirrha*, with a scaffold N50 length of 733.65 Kb, and annotated 21,606 protein-coding genes. The ncRNAs annotation, especially for miRNA, could help us study the expression regulation of coding genes in the future. Comparative genomics analysis indicated that the large *C. quinquecirrha* genome was mainly due to non-coding region expansion. Codon usage analysis indicated that *C. quinquecirrha* tends to use low-energy codons in the protein-coding genes. Furthermore, results demonstrated that *C. quinquecirrha* has a relatively faster evolution rate than sea anemones but slower evolution rate than the species in Hydra. Phylogenetic results indicated that *A. aurita* and *N. nomurai* are more closely related to each other than to *C. quinquecirrha*, with divergence between their common ancestor and *C. quinquecirrha* occurring 475.7 Mya. Simple morphological considerations, several previous studies got different phylogenetic relationships among these species. Our study analyzed the phylogeny by the whole-genome data could help us better understand the evolution and their relationships in Cnidaria.

## Data Availability Statement

All raw sequencing reads were deposited in the Sequence Read Archive (SRA) database of National Center for Biotechnology Information (NCBI) with accession number of PRJNA555711. Besides, the genome assembly (https://www.ncbi.nlm.nih.gov/assembly/GCA_012295145.1) and transcript assembly (https://www.ncbi.nlm.nih.gov/nuccore/GILX00000000) were also uploaded to the NCBI database. All the other data, including genome assembly file, gff file, protein-coding genes, and the *de novo* assembled transcripts with the RNA-seq data, were uploaded to the DRYAD database (https://doi.org/10.5061/dryad.brv15dv6c).

## Author Contributions

YL and XG conceived and supervised the project and revised the manuscript. WX and YL collected samples. WX and Hao Li performed bioinformatics analyses. WX and Hao Li wrote the manuscript. WC, Hon Li, and YM revised the manuscript. All authors have read and approved the final manuscript.

## Conflict of Interest

The authors declare that the research was conducted in the absence of any commercial or financial relationships that could be construed as a potential conflict of interest.

## Footnotes

1 https://github.com/Nextomics/NextDenovo

2 http://www.repeatmasker.org/RepeatModeler/

3 http://www.timetree.org
