# Supplementary material for: High-Quality Genome Assembly of Chrysaora quinquecirrha Provides Insights Into the Adaptive Evolution of Jellyfish
Source: Front Genet. 2020 Jun 4;11:535. doi: 10.3389/fgene.2020.00535 (PMC7287180; doi:10.3389/fgene.2020.00535)
Supplement: Supplementary file 1 [file Data_Sheet_1.pdf]

Supplementary Material

Supplementary Tables

Table S1. The statistics of sequencing reads on Illumina platform. These data were produced by short insert library. Results are shown for filtered reads. Sequencing depth was calculated by assembled genome size.

Library ID	Read Pairs	Total bases	Sequencing strategy	Sequencing depth (X)
NGS-L1	171,522,496	51,456,748,800	PE150	156.25

Table S2. The statistics of sequencing reads on Nanopore platform. Only reads with quality value Q > 7 were used.

Library ID	Average length	N50 length	Read number	Total bases
TGS-L1	23,259	29,300	1,305,874	30,373,512,861
TGS-L2	22,923	27,729	2,213,623	50,744,961,441
Total/Average	23,091	28,515	3,519,497	81,118,474,302

Table S3. Comparison of genome assembly quality.

Species	Genome source	Genome size	Scaffold N50	Contig N50	Total scaffold number	Total contig number	Gene number	BUSCO
Hydra vulgaris	NCBI	852,170,992	96,317	10,112	20,916	126,669	20,055	83.70%
Stylophora pistillata	NCBI	400,120,318	457,453	20,604	5,688	37,615	24,833	88.00%
Acropora digitifera	NCBI	447,497,157	483,559	10,915	2,421	54,401	26,073	74.40%
Nematostella vectensis	NCBI	356,613,585	472,588	19,835	19,835	59,149	24,780	91.40%
Exaiptasia pallida	NCBI	256,132,296	442,145	14,401	4,312	29,750	22,509	87.10%
Aurelia aurita	NCBI	757,170,055	1,042,981	33,962	2709	19,288	27,044	84.10%
Renilla muelleri	GigaDB	172,160,214	70,522	64.781	4925	5,196	23,671	87.70%
Nemopilema nomurai	NCBI	213,621,014	2,711,397	849,297	251	1,463	19,525	86.90%
Clytia hemisphaerica	marimba	445,210,140	366,311	3,861	7,644	187,243	25,087	86.40%
Mnemiopsis leidyi	nhgri	155,865,547	187,314	11,936	5,100	24,884	16,548	77.30%

H. vulgaris: https://www.ncbi.nlm.nih.gov/assembly/GCF_000004095.1; *S. pistillata*: https://www.ncbi.nlm.nih.gov/assembly/GCF_002571385.1; *A. digitifera*: https://www.ncbi.nlm.nih.gov/assembly/GCF_000222465.1; *N. vectensis*: https://www.ncbi.nlm.nih.gov/assembly/GCF_000209225.1; *E. pallida*: https://www.ncbi.nlm.nih.gov/assembly/GCF_001417965.1; *A. aurita*: https://www.ncbi.nlm.nih.gov/assembly/GCA_004194415.1; *R. muelleri*: http://gigadb.org/dataset/100565; *N. nomurai*: https://www.ncbi.nlm.nih.gov/assembly/GCA_003864495.1; *C. hemisphaerica*: http://marimba.obs-vlfr.fr/organism/Clytia/hemisphaerica; *M. leidyi*: https://research.nhgri.nih.gov/mnemiopsis.

Table S4. Assembly quality evaluation using BUSCO.

Dataset	Library	Percentage (%)
Genome	Eukaryota	C:79.6%[S:66.7%,D:12.9%],F:8.3%,M:12.1%,n:303
	Metazoa	C:78.8%[S:63.9%,D:14.9%],F:5.0%,M:16.2%,n:978
CDS	Eukaryota	C:77.2%[S:66.3%,D:10.9%],F:10.2%,M:12.6%,n:303

	Metazoa	C:73.3%[S:61.2%,D:12.1%],F:7.8%,M:18.9%,n:978
Protein	Eukaryota	C:77.9%[S:66.3%,D:11.6%],F:8.3%,M:13.8%,n:303
	Metazoa	C:72.9%[S:61.0%,D:11.9%],F:7.7%,M:19.4%,n:978

Note: Complete BUSCOs (C); Complete and single-copy BUSCOs (S); Complete and duplicated BUSCOs (D); Fragmented BUSCOs (F); Missing BUSCOs (M); Total BUSCO groups searched (n).

Table S5. The statistics of RNA sequencing reads on Illumina platform. These data were produced by short insert library. Results are shown for filtered reads.

Library ID	Read pairs	Tissue	Sequencing strategy	Total bases
T1	28,556,004	muscle	PE150	7,995,681,120
T2	18,864,370	muscle	PE150	5,282,023,600
Total	47,420,374	-	-	13,277,704,720

Table S6. The statistics of *de novo* transcript assembly by Bridger software.

Term	Length (bp)	Number
N90	336	71,913
N80	634	44,269
N70	1,207	30,129
N60	1,775	21,748
N50	2,305	15,661
Max length (bp)	31,711	-
Total size (bp)	123,770,934	-
Total number (>100 bp)	-	120,198
Total number (>10 kb)	-	220

Table S7. Transcript assembly evaluation using BUSCO software.

Library	Percentage (%)
Eukaryota	C:99.6%[S:34.3%,D:65.3%],F:0.3%,M:0.1%,n:303
Metazoa	C:96.0%[S:32.2%,D:63.8%],F:1.0%,M:3.0%,n:978

Note: Complete BUSCOs (C); Complete and single-copy BUSCOs (S); Complete and duplicated BUSCOs (D); Fragmented BUSCOs (F); Missing BUSCOs (M); Total BUSCO groups searched (n).

Table S8. Mapping ratio of Illumina short reads on the assembled genome. PE mapped represents reads mapped to genome as read pairs, SE mapped represents reads mapped to genome as single reads.

Term	# Mapped reads	Mapped ratio (%)
PE mapped	248,672,844	73.52%
SE mapped	4,727,992	1.40%
Total mapped	273,209,877	79.76%

Table S9. Mapping ratio of transcripts in genome assembly.

Total transcripts	Mapped number	Mapping ratio (%)
120,198	85,556	71.18

Table S10. The statistics of annotated repetitive sequences in *C. quinquecirrha* genome.

Software	Repeat Size (bp)	% of genome
TRF	34,152,943	10.139838

Repeatmasker	11,940,847	3.545178
Proteinmasker	13,648,690	4.052228
RepeatModeler	121,407,295	36.045219
Total	149,862,365	44.493388

Table S11. The statistics of annotated protein-coding genes in *C. quinquecirrha* genome. GN, gene number; AML, average mRNA length; TEN, total exon number; AEL, average exon length; AEN, average exon number; TIL, total intron length.

GN	AML	TEN	AEL	AEN	TIL
21,606	6,014.50	131,349	248.55	6.08	97,302,196

Table S12. The statistics of gene families among species.

Species	Gene number	Gene in family	Unclustered genes	Family number	Unique families
S. pistillata	24,846	21,052	3,794	13,948	655
E. pallida	22,509	19,057	3,452	12,878	534
R. muelleri	23,671	19,951	3,720	8,828	816
A. aurita	27,044	19,340	7,704	11,773	1,366
A. digitifera	26,073	21,559	4,514	13,366	631
C. quinquecirrha	21,606	17,370	4,236	9,631	728
H. vulgaris	20,055	16,673	3,382	8,595	653
E. granulosus	11,319	5,676	5,643	4,366	339
N. vectensis	24,780	19,184	5,596	12,794	887
N. nomurai	19,525	16,364	3,161	11,217	477

Table S13. The KEGG enrichment analysis of specific gene family in all jellyfish species.

Map Number	Pathway	Count	*P*-value
map04340	Hedgehog signaling pathway	3	0.001342
map05217	Basal cell carcinoma	3	0.001521
map04350	TGF-beta signaling pathway	2	0.012341
map05200	Pathways in cancer	4	0.035948
map04360	Axon guidance	2	0.036715
map04390	Hippo signaling pathway	2	0.036715

Table S14. The calibration data used in divergence time analysis.

Node	Estimated Time	Range	Reference (DOI)
S. pistillata and *A. digitifera*	250 MYA	248 - 253 MYA	10.1016/j.ympev.2011.10.008; 10.2307/41317044; 10.1002/ece3.527
A. aurita and *C. quinquecirrha*	451 MYA	CI: n/a	10.1016/j.ympev.2011.10.008
N. vectensis and (*S. pistillata* and *A. digitifera*)	569 MYA	539 - 600 MYA	10.1016/j.cub.2015.09.066; 10.1111/ede.12168; 10.1016/j.ympev.2015.05.013; 10.1016/j.ympev.2011.10.008
H. vulgaris and (*A. aurita* and *C. quinquecirrha*)	581 MYA	556 - 607 MYA	10.1016/j.ympev.2015.05.013; 10.1016/j.ympev.2011.10.008;
(*N. vectensis*, *S. pistillata* and *A. digitifera*) and (*H. vulgaris*, *A.*	617 MYA	CI: 540 - 667 MYA	10.1073/pnas.0503660102; 10.1073/pnas.0401670101;

aurita and *C. quinquecirrha*)			10.1111/ede.12168; 10.1093/icb/icm071; 10.1016/j.cub.2015.09.066; 10.1016/j.ympev.2015.05.013; 10.1098/rstb.2007.2233; 10.1016/j.ympev.2011.10.008
E. granulosus and (*N. vectensis*, *S. pistillata*, *A. digitifera*, *H. vulgaris*, *A. aurita* and *C. quinquecirrha*)	824 MYA	CI: 611 - 1035 MYA	10.1073/pnas.0503660102; 10.1073/pnas.0401670101; 10.1038/srep04127; 10.2307/3070910; 10.1016/j.cub.2015.09.066; 10.1098/rstb.2007.2233; 10.1111/ede.12168; 10.1073/pnas.1110633108; 10.2307/2640769; 10.1016/S0022-5193(03)00057-2; 10.1186/1471-2148-4-2

Table S15. The GO enrichment analysis of expanded gene families in all jellyfish species.

GO ID	GO Term	GO Class	*P*-value
GO:0008146	sulfotransferase activity	MF	1.49E-24
GO:0003676	nucleic acid binding	MF	8.60E-21

Table S16. The KEGG enrichment analysis of expanded gene families in all jellyfish species.

Map Number	Pathway	Count	*P*-value
map00062	Fatty acid elongation	19	1.96E-05
map05206	MicroRNAs in cancer	28	0.000138492
map05222	Small cell lung cancer	22	0.000159139
map00930	Caprolactam degradation	17	0.000257858
map04320	Dorso-ventral axis formation	12	0.000264635
map00362	Benzoate degradation	17	0.000293621
map04724	Glutamatergic synapse	17	0.000427916
map00627	Aminobenzoate degradation	17	0.000612208
map04142	Lysosome	21	0.001330432
map00410	beta-Alanine metabolism	17	0.00217962
map00650	Butanoate metabolism	17	0.002633381
map00071	Fatty acid degradation	18	0.002908315
map00380	Tryptophan metabolism	17	0.003778665
map04330	Notch signaling pathway	12	0.005299582
map00640	Propanoate metabolism	17	0.005758856
map05216	Thyroid cancer	11	0.006118495
map00984	Steroid degradation	3	0.009528671
map00280	Valine, leucine and isoleucine degradation	17	0.009872379
map01212	Fatty acid metabolism	17	0.013110729
map05030	Cocaine addiction	10	0.016803431
map03018	RNA degradation	21	0.017749821
map04512	ECM-receptor interaction	20	0.018005727

map05219	Bladder cancer	10	0.020359278
map05200	Pathways in cancer	38	0.020915917
map01040	Biosynthesis of unsaturated fatty acids	5	0.022660713
map05223	Non-small cell lung cancer	10	0.031605665
map00310	Lysine degradation	17	0.033132069
map05132	Salmonella infection	13	0.036615923
map05218	Melanoma	10	0.03713848
map03013	RNA transport	20	0.049512577

Table S17. The codon usage analysis based on CondonW.

Species	T3s	C3s	A3s	G3s	CAI	CBI	Fop
S. pistillata	0.38	0.2605	0.3747	0.26	0.112	0.045	0.43
E. pallida	0.399	0.247	0.4111	0.225	0.122	0.05	0.44
R. muelleri	0.41	0.2182	0.4219	0.238	0.121	0.029	0.42
A. aurita	0.376	0.2389	0.4216	0.255	0.115	0.029	0.42
A. digitifera	0.38	0.2617	0.3698	0.263	0.113	0.049	0.43
C. quinquecirrha	0.410	0.2233	0.4317	0.231	0.123	0.03	0.43
H. vulgaris	0.511	0.1463	0.4905	0.155	0.135	-0.01	0.41
E. granulosus	0.35	0.3251	0.276	0.287	0.096	0.042	0.42
A.queenslandica	0.364	0.2561	0.3819	0.257	0.107	0.044	0.424
M. leidyi	0.341	0.3161	0.3341	0.285	0.111	0.078	0.45
N. vectensis	0.328	0.3202	0.3182	0.3	0.099	0.033	0.42
N. nomurai	0.347	0.2849	0.3583	0.294	0.11	0.047	0.43
Species	Nc	GC3s	GC	L_sym	L_aa	Gravy	Aromo
S. pistillata	55.9	0.4	0.44	12,394,983	12,829,521	-0.3895	0.084224
E. pallida	54.1	0.36	0.419	10,970,204	11,368,571	-0.41696	0.085453
R. muelleri	53.8	0.35	0.413	11,233,151	11,632,190	-0.49698	0.077992
A. aurita	53.4	0.37	0.412	11,827,819	12,308,923	-0.42237	0.089275
A. digitifera	56	0.4	0.44	11,233,718	11,627,719	-0.36714	0.084673
C. quinquecirrha	52	0.34	0.405	10,471,744	10,843,663	-0.46888	0.082877
H. vulgaris	44.9	0.22	0.339	8,181,216	8,454,384	-0.32607	0.095167
E. granulosus	58.7	0.49	0.494	5,106,193	5,277,910	-0.28106	0.078778
A.queenslandica	54.6	0.40	0.423	8,797,823	9,215,299	-0.21025	0.097323
M. leidyi	56.9	0.46	0.458	7,383,239	7,637,068	-0.40296	0.081944
N. vectensis	59.1	0.48	0.474	7,988,803	8,293,994	-0.30605	0.087586
N. nomurai	56.7	0.44	0.45	9,159,681	9,480,074	-0.42319	0.081823

Table S18. The codon usage of protein-coding genes in *C. quinquecirrha*.

Amino acid	Codon	Frequency	Number
Phe	UUU	1.186	266,166
	UUC	0.814	182,706
Leu	UUA	1.088	172,096
	UUG	1.409	222,871
	CUU	1.336	211,197
	CUC	0.547	86,438

	CUA	0.688	108,716
	CUG	0.932	147,412
Ser	UCU	1.320	289,077
	UCC	0.820	179,708
	UCA	0.860	188,365
	UCG	1.000	250,361
Pro	CCU	1.602	257,413
	CCC	0.778	124,936
	CCA	0.833	133,822
	CCG	0.787	126,405
Tyr	UAU	1.224	180,633
	UAC	0.503	74,280
Ter	UAA	1.671	246,562
	UAG	0.529	78,063
Cys	UGU	1.190	175,664
	UGC	0.883	130,324
Ter	UGA	1.261	139,144
Trp	UGG	1.814	200,209
His	CAU	0.426	47,066
	CAC	0.499	55,017
Gln	CAA	1.167	171,575
	CAG	1.700	249,933
Arg	CGU	0.578	84,928
	CGC	0.556	81,795
	CGA	1.366	217,295
	CGG	0.600	95,422
Ile	AUU	1.650	262,509
	AUC	0.385	61,225
	AUA	1.209	198,485
Met	AUG	0.791	129,771
Thr	ACU	1.110	14,116
	ACC	0.611	7,766
	ACA	1.280	16,278
	ACG	1.350	171,441
Asn	AAU	0.650	82,452
	AAC	1.220	273,187
Lys	AAA	0.780	174,754
	AAG	1.267	361,858
Ser	AGU	0.733	209,570
	AGC	1.258	532,808
Arg	AGA	0.742	314,239
	AGG	1.377	441,374
Val	GUU	0.623	199,567
	GUC	1.369	526,310
	GUA	0.631	242,630
	GUC	1.105	125,845

Ala	GCU	0.895	101,899
	GCC	1.000	121,558
	GCA	0.753	72,198
	GCG	0.425	40,745
Asp	GAU	0.926	88,711
	GAC	0.304	29,099
Glu	GAA	2.630	252,073
	GAG	0.962	92,166
Gly	GGU	1.182	165,920
	GGC	1.559	218,919
	GGA	0.766	107,479
	GGG	0.493	69,272

Table S19. The relative evolution rate of species employed by LINTRE.

Outgroup	Ingroup1	Ingroup2	bA	bB	delta	Z score	CP
E. granulosus	*C. quinquecirrha*	*S. pistillata*	0.375811	0.524235	0.148424	19.057774	99.96%
E. granulosus	*C. quinquecirrha*	*E. pallida*	0.387301	0.514623	0.127322	16.245096	99.96%
E. granulosus	*C. quinquecirrha*	*R. muelleri*	0.497607	0.508538	0.010931	1.295492	80.30%
E. granulosus	*C. quinquecirrha*	*A. aurita*	0.174391	0.222536	0.048146	7.835847	99.96%
E. granulosus	*C. quinquecirrha*	*A. digitifera*	0.444085	0.501671	0.057586	6.839982	99.96%
E. granulosus	*C. quinquecirrha*	*H. vulgaris*	0.455097	0.392384	0.062713	7.824449	99.96%
E. granulosus	*C. quinquecirrha*	*N. vectensis*	0.329763	0.445399	0.115637	16.677930	99.96%
E. granulosus	*C. quinquecirrha*	*N. nomurai*	0.193754	0.246914	0.053160	8.820108	99.96%

Table S20. The relative evolution rate of species employed by MEGA.

Outgroup	Ingroup1	Ingroup2	Identical	Ingroup1 specific	Ingroup2 specific	Chi-score	*P*-value
E. granulosus	*C. quinquecirrha*	*R. muelleri*	47,702	13,878	13,663	1.68	0.19514
E. granulosus	*C. quinquecirrha*	*A. aurita*	50,205	6,909	6,017	61.56	0.000001
E. granulosus	*C. quinquecirrha*	*A. digitifera*	45,932	13,650	12,542	46.87	0.000001
E. granulosus	*C. quinquecirrha*	*H. vulgaris*	47,587	11,266	12,473	61.37	0.000001
E. granulosus	*C. quinquecirrha*	*N. vectensis*	50,644	13,293	10,697	280.92	0.000001
E. granulosus	*C. quinquecirrha*	*N. nomurai*	57,391	8,585	7,466	78.01	0.000001
E. granulosus	*C. quinquecirrha*	*S. pistillata*	52,011	15,452	12,259	367.91	0.000001
E. granulosus	*C. quinquecirrha*	*E. pallida*	50,596	14,797	12,119	266.45	0.000001
